# Development, Reliability, and Structural Validity of the Scale for Knowledge, Attitude, and Practice in Ethics Implementation Among AI Researchers: Cross-Sectional Study

**DOI:** 10.2196/42202

**Published:** 2023-10-26

**Authors:** Xiaobo Zhang, Ying Gu, Jie Yin, Yuejie Zhang, Cheng Jin, Weibing Wang, Albert Martin Li, Yingwen Wang, Ling Su, Hong Xu, Xiaoling Ge, Chengjie Ye, Liangfeng Tang, Bing Shen, Jinwu Fang, Daoyang Wang, Rui Feng

**Affiliations:** 1 Children's Hospital of Fudan University Shanghai China; 2 School of Philosophy Fudan University Shanghai China; 3 School of Computer Science Fudan University Shanghai China; 4 School of Public Health Fudan University Shanghai China; 5 Department of Paediatrics Faculty of Medicine The Chinese University of Hong Kong Hong Kong SAR China; 6 Shanghai Hospital Development Center Shanghai China

**Keywords:** medical artificial intelligence, ethics implementation, Knowledge-Attitude-Practice model, reliability, validity, measure, artificial intelligence, development, attitude, ethics

## Abstract

**Background:**

Medical artificial intelligence (AI) has significantly contributed to decision support for disease screening, diagnosis, and management. With the growing number of medical AI developments and applications, incorporating ethics is considered essential to avoiding harm and ensuring broad benefits in the lifecycle of medical AI. One of the premises for effectively implementing ethics in Medical AI research necessitates researchers' comprehensive knowledge, enthusiastic attitude, and practical experience. However, there is currently a lack of an available instrument to measure these aspects.

**Objective:**

The aim of this study was to develop a comprehensive scale for measuring the knowledge, attitude, and practice of ethics implementation among medical AI researchers, and to evaluate its measurement properties.

**Methods:**

The construct of the Knowledge-Attitude-Practice in Ethics Implementation (KAP-EI) scale was based on the Knowledge-Attitude-Practice (KAP) model, and the evaluation of its measurement properties was in compliance with the COnsensus-based Standards for the selection of health status Measurement INstruments (COSMIN) reporting guidelines for studies on measurement instruments. The study was conducted in 2 phases. The first phase involved scale development through a systematic literature review, qualitative interviews, and item analysis based on a cross-sectional survey. The second phase involved evaluation of structural validity and reliability through another cross-sectional study.

**Results:**

The KAP-EI scale had 3 dimensions including knowledge (10 items), attitude (6 items), and practice (7 items). The Cronbach α for the whole scale reached .934. Confirmatory factor analysis showed that the goodness-of-fit indices of the scale were satisfactory (*χ^2^*/*df* ratio:=2.338, comparative fit index=0.949, Tucker Lewis index=0.941, root-mean-square error of approximation=0.064, and standardized root-mean-square residual=0.052).

**Conclusions:**

The results show that the scale has good reliability and structural validity; hence, it could be considered an effective instrument. This is the first instrument developed for this purpose.

## Introduction

The expression “artificial intelligence” (AI) was introduced by John McCarthy, and the official birth of AI as a field of research was unanimously considered at the Dartmouth Conference in 1956 [1]. AI has been defined as “the machine simulation of human mental reasoning, decision making, and behavior” [2]. The increased power of computing, storage capacity expansion, and compilation of big medical data helped the AI implementation surge in medical practice and research [2]. Medical AI is currently used in various medical fields such as medical image analysis, disease screening and prediction, clinical decision support, surgical robotics, health management, virtual medical assistants, and aiding in screening drug targets [3-7]. However, while the rapid development of AI brings higher efficiency, accuracy, and convenience to medicine and health care, it is also accompanied by many risks and challenges. Such issues are related to the intrusion of AI algorithms into the privacy and intimacy of people under investigation, enormous deficits of informed consent detected in AI research processes, and shirking liability in medical malpractice where AI is applied in decision-making [8-10]. There is now a growing consensus among experts that implementing ethics in medical AI research is crucial [11].

National and international research institutions have put forward several principles or guidelines for ethical governance of medical AI. The guidelines of the *Ethics and Governance of Artificial Intelligence for Health* (World Health Organization, 2021) [12] contains a set of recommendations to ensure that the governance of AI holds all stakeholders accountable and responsive to end users, and *The* *New Generation of Ethical Norms of Artificial Intelligence* (Ministry of Science and Technology of China, 2021) [13] and *The* *Guidelines of Strengthening Governance over Ethics in Science, Technology* (General Office of the State Council of China, 2022) [14] raise some similar principles of ethics governance in the development and application of AI. Meanwhile, the Data Governance Act (European Parliament, 2020) [15] suggests certain technical instruments to ensure the preservation of protection, privacy, and confidentiality in the transfer, reuse, and recovery of data by third parties, and the Artificial Intelligence Act (European Parliament, 2021) [16] establishes a European Artificial Intelligence Committee to ensure compliance with the implementation and enforcement of the regulations and encourage the exchange of best practices. There are also several strategies for implementing ethics into medical AI research. One approach is to consider ethics at the design and requirement capture stage by embedding ethical values into the application using methodologies such as Value-Sensitive-Design [17] and Values in Motion Design [18]. Others consist of coding ethics in the operating system [19], embedding ethics principles in the algorithm [20], and monitoring and evaluating the applications [21].

Implementing ethics is essentially motivated by the need to gain the trust of the patients, the implementor being the researcher [22]. Successful ethics engagement requires the ethical competence of stakeholders as well as the intention to comply with corresponding values. The prerequisite is that the researchers master relevant ethical knowledge, agree with ethical values, and then behave as expected [23]. Currently published ethics implementation evaluation mainly focuses on better patient outcomes [22,24], reporting the safety, equity, cost-effectiveness, privacy, clear professional responsibilities, autonomy, justice, and fairness in AI development and implementation [22,24-27], conducted through checklists, questionnaires, or stakeholders' consultations to promote careful design and execution of medical AI research, and to assess the ethical and social implications of AI implementations [28-30]. We frequently fail to consider the perception of ethics implementation among medical AI researchers, which serves as the fundamental driving force for ensuring ethical practices. To address this gap, it is crucial to develop tools that comprehensively measure AI researchers' knowledge, attitudes, and practices of ethics implementation.

The Knowledge-Attitude-Practice (KAP) model is widely used in medical research as the most commonly used model [31-33], proposed that knowledge was the basis of behavior change, and attitude and practice are the driving force of behavior change. This study aimed to develop a scale based on the KAP model for measuring the perception of ethics implementation among medical AI researchers and evaluate the reliability and structural validity of the scale. Our hypothesis is that the scale is well-designed and has good measurement properties.

## Methods

### Study Design

The study was conducted in 2 phases. Item generation, expression refinement, and item analysis were involved in the first phase through systematic literature reviews, qualitative analysis, and item analysis based on a cross-sectional study. In the second phase, another cross-sectional survey was conducted to test the measurement properties of the developed scale.

### Procedures and Participants

#### Phase 1-1: Item Generation and Cognitive Interview

The KAP model was used as the conceptual framework to define the construct to be measured. Based on the model, knowledge is composed of scientific knowledge, local knowledge, tacit knowledge, and self-reflective knowledge [34]. Attitude referred to a positive or negative option of objective evaluation [35]. Practice included regular activities influenced by widely shared beliefs [36]. The initial step involved systematic literature retrieval to gather guidelines, expert consensus, practice standards, and norms referring to the implementation of ethics in AI research. A librarian working in the hospital library provided valuable assistance during this process (see Multimedia Appendix 1). Then, a focus group interview was conducted, consisting of 10 experts (2 medical ethics professors, a sociology professor, 3 AI professors, and 4 medical professors proficient in medical AI implementation research). They expressed their opinions on the following issues: (1) what is your understanding of implementing ethics in medical AI research? (2) What knowledge do you think medical AI researchers should master to help implement ethics? (3) What are your perceptions of implementing ethics in medical AI research? (4) How do you implement ethics in medical AI research? Eventually, relevant contents from various bodies of literature and interviews were extracted and classified in accordance with the items.

All the generated items were sent to another 10 experts (including medical ethics professors, sociology professors, AI professors, and medical professors) for consultation. Item deletion and revision were applied in accordance with the findings from 3 rounds of expert consultation. After that, the first draft was formed. Eight people (including AI researchers, health care workers, and health information managers) were invited to complete the first draft scale and then interviewed with the following questions: (1) was each item clearly expressed without ambiguity? If no, please identify the unclear or ambiguous expressions. (2) Were there any items difficult to understand? If yes, please identify the difficulties and if not, please try to explain each item in your own words. (3) What were your reasons for each of your answers? (4) What else is needed to be added? Language readability of each point was modified in accordance with the comments. The time that each person spent completing the questionnaire was also recorded. The final draft was a 5-point Likert scale with 3 dimensions. The responses for the dimension of knowledge ranged from “not familiar at all (1 point)” to “extremely familiar (5 points),” that for attitudes ranged from “strongly disagree (1 point)” to “strongly agree (5 points),” and that for practice ranged from “never (1 point)” to “always (5 points).” Reverse scoring was performed for the items running in the opposite direction. Face validity was calculated by scoring with a 4-point scale [37] (1=not relevant, 2=unable to assess or need much revision, 3=relevant but need minor revision, and 4=very relevant and succinct) with the other 52 participants. The inclusion criteria were AI developers, AI algorithm engineers, or AI implementation researchers with relevant experiences of more than 5 years.

#### Phase 1-2: Item Analysis

A cross-sectional survey was conducted on June 25, 2022, and ended on July 31, 2022. Considering that the amount of medical AI researchers is relatively small, snowball sampling [38-40] was used to enrich survey samples in the cross-sectional study. Researchers of medical AI development or medical AI application were eligible to serve as participants if they had taken part in more than 1 medical AI research project. Potential participants were excluded if none of the projects they were involved in had been finished; if the time they spent completing the survey was too short or too long (time<mean-SD or time>mean+SD), if their answers to demographic questions were illogical, and if their answers to all items were the same. At first, 6 medical AI researchers who had previously worked on medical AI research projects with our hospital were identified. They were from hospitals, the department of computer science at a certain university, and 3 computer programming companies across the regions of North China, East China, and Northeast China. Initially, they were invited to complete the questionnaire, and then they were asked to send the QR code or link of the blank questionnaire to their colleagues or research partners who were eligible and might be willing to be recruited. Subsequent participants repeated the abovementioned procedure to recruit other potential participants until the required sample size was achieved. Ideally, each participant was asked to invite 3-5 eligible individuals to join the study.

We distributed an electronic questionnaire including participants' demographics and the final draft by Wenjuanxing [41], a professional and widely used website for conducting surveys in China. Participants could scan the QR code using their cell phones or log in on their computers to access and complete the questionnaire. The purpose of the survey and answering instructions were described on the first page of the web-based questionnaire. The participants were suggested to complete the questionnaire within 5 to 10 minutes. The time limit was set on the basis of the actual time spent on the questionnaire recorded in the first phase of the study. There is also a limit on respondents’ IP addresses to avoid multiple enrollments. A reminder for checking blank answers was set to block the submission of unfinished questionnaires.

#### Phase 2: Testing Reliability and Structural Validity

Another cross-sectional survey was conducted on February 20, 2023, and ended on April 26, 2023, for testing the reliability and structural validity of the scale, following the COnsensus-based Standards for the selection of health status Measurement INstruments (COSMIN) reporting guideline [42]. Snowball sampling was adopted again. The inclusion and exclusion criteria were the same as those in the first cross-sectional study. The sample-to-item ratio is used to decide the sample size. The sample size was estimated at 15 to 20 participants per item in the first survey [43]. As there were 23 items in the developed scale, a sample size of 345 to 460 participants was required. Paper questionnaires were distributed by trained investigators employed at each survey site.

### Statistical Analysis

Excel 365 for Windows (Microsoft Corp) was used to establish a database. Data were analyzed using SPSS (version 25.0; IBM Corp) and AMOS (version 24.0; IBM Corp) for Windows.

Descriptive statistics are used to show the characteristics of the participants involved in the cross-sectional studies. Item analysis was conducted as described previously [44] by calculating the following: (1) item discrimination: after ranking the participants by their total score on items, we selected those from the top 27% and the bottom 27% and ran an independent *t* test to determine whether each item could significantly distinguish the 2 groups. The item that failed to do so or whose *t* value was <3 would be removed. (2) Item correlation: we inspected the correlation matrix between items and scale and removed the item whose correlation coefficient was less than 0.40. (3) Item homogeneity: we measured the Cronbach α coefficient of the scale first and inspected the change in value by deleting 1 item at a time. The item would be removed if the changeable value was significantly higher than the original one, the absolute value of factor loading was less than 0.4, or the value of community was less than 0.6.

A test of structural validity was performed using exploratory factor analysis (EFA) and confirmatory factor analysis (CFA). The Kaiser-Meyer-Olkin (KMO) and Bartlett tests were used to determine whether our data were suitable for EFA. A Bartlett score of <0.05 and a KMO score of ≥0.7 were considered appropriate. The Varimax oblique rotation method was applied to extract the factor loadings [45]. As the KAP model is composed of 3 dimensions, the third-order CFA model was used to establish the scale’s construct validity. To assess the model’s fitness, the following absolute and incremental fit indices were used: (1) root-mean-square error of approximation (RMSEA), with 0.08 as a cutoff for poor-fitting models; (2) standardized root-mean-square residual (SRMR), where a value of less than 0.08 is generally considered a good fit; (3) comparative fit index (CFI) ranging between 0.0 and 1.0, where values closer to 1.0 indicate good fit (CFI≥0.90); and (4) Tucker Lewis Index (TLI), also ranging between 0.0 and 1.0, where TLI≥0.9 indicates a good fit [45-48].

For face validity, the item-level content validity index (I-CVI) was computed as the number of experts assigning a scoring of 4 or 3 for each item divided by the total number of experts. Similarly, the average scale–level CVI (S-CVI/Ave) was calculated using the number of items that achieved a scoring of 4 or 3 divided by the total number of items. Interrater reliability (IRR) was calculated using the total number of items scoring in agreement divided by the total number of items. An I-CVI of ≥0.8, S-CVI/Ave of ≥0.9, and IRR of >0.7 were considered acceptable [49,50].

For the reliability of the scale, a Cronbach α of ≥.7 was used as the reference. Both values for split-half reliability and test-retest reliability of ≥0.7 were considered acceptable.

### Ethical Considerations

The study was conducted in accordance with the guidelines of the Declaration of Helsinki and approved by the Research Ethics Board of the Children's Hospital of Fudan University (2022-52).

## Results

### Sample Characteristics

A total of 306 responses were received in the first survey and 48 questionnaires were excluded (5 with illogical answers about date of birth, 23 with an answering time of <8.21 (SD 4.18) minutes, and 9 had the same answers to all items). Finally, 269 questionnaires were included in the analysis of the first survey. Similarly, 481 responses were received in the second survey with 11 questionnaires excluded for having the same answers to all items. The characteristics of the participants are shown in Table 1.

**Table 1:** Characteristics of participants in the first and second surveys.

Characteristic		Participants in the first survey, n (%)	Participants in the second survey, n (%)
**Gender**			
	Male	196 (72.9)	368(78.3)
	Female	73 (27.1)	102 (21.7)
**Related working experience**			
	<3 MAI^a^ research projects	137 (50.9)	231 (49.1)
	3-5 MAI research projects	102 (37.9)	179 (38.1)
	6-10 MAI research projects	22 (8.2)	43 (9.1)
	>10 MAI research projects	8 (3.0)	17 (3.6)
**Education level**			
	Bachelor's degree	108 (40.2)	185 (39.3)
	Master's degree	101 (37.5)	171 (36.3)
	Doctoral degree	60 (22.3)	114 (24.3)
**Occupation**			
	Medical staff	60 (22.3)	134 (28.5)
	Health information manager	65 (24.2)	148 (31.5)
	AI research and development Engineer or algorithm engineer	68 (25.3)	117 (24.8)
	Medical student	11 (4.1)	0 (0)
	Computer science student	65 (24.2)	71 (15.1)

^a^MAI: medical artificial intelligence.

### Development of the Knowledge-Attitude-Practice in Ethics Implementation Scale

In total, 25 items and 3 dimensions (9 for knowledge, 7 for attitude, and 9 for practice) were generated first. Two identical items were combined, 1 miscellaneous item was split, 8 items were revised for obscure expression, and 3 items were added as suggested by the experts during expert consultation. The experts for content validity assessment also proposed wording amendments in the practice dimension, such that the same content is expressed in 2 ways from the perspective of both research leaders or primary researchers and research participants. The final draft (see Multimedia Appendix 2) had 28 items (F1- F28). According to the results of item analysis, 5 items (F14, F17, F19, F20, and F21) were removed for the suboptimal absolute value of the critical ratio (CR), factor loading, correlation coefficient, and Cronbach α coefficient (after the change), as shown in Table 2. The final version of the Knowledge-Attitude-Practice in Ethics Implementation (KAP-EI) scale is presented in Multimedia Appendix 3 with 23 items.

**Table 2:** Results of item analysis (N=28; Cronbach α=.904).

Item	Item discrimination, critical ratio (*P* value)	Item correlation, *r* (*P* value)	Item homogeneity				Substandard items, n		Comments
			Cronbach α (after the change)	Value of community	Value of factor loading				
F1	8.567 (<.001)	0.553 (<.001)	0.901	0.507	0.612	0		Reserved	
F2	11.741 (<.001)	0.697 (<.001)	0.898	0.713	0.774	0		Reserved	
F3	12.84 (<.001)	0.716 (<.001)	0.898	0.764	0.790	0		Reserved	
F4	13.995 (<.001)	0.738 (<.001)	0.897	0.792	0.820	0		Reserved	
F5	16.119 (<.001)	0.792 (<.001)	0.896	0.847	0.868	0		Reserved	
F6	13.874 (<.001)	0.759 (<.001)	0.897	0.809	0.846	0		Reserved	
F7	16.168 (<.001)	0.782 (<.001)	0.896	0.848	0.856	0		Reserved	
F8	15.388 (<.001)	0.754 (<.001)	0.897	0.817	0.831	0		Reserved	
F9	14.848 (<.001)	0.767 (<.001)	0.896	0.824	0.843	0		Reserved	
F10	14.326 (<.001)	0.755 (<.001)	0.897	0.726	0.813	0		Reserved	
F11	6.078 (<.001)	0.403 (<.001)	0.903	0.690	0.724	0		Reserved	
F12	3.896 (<.001)	0.332^a^ (<.001)	0.904	0.784	0.838	1		Reserved	
F13	5.792 (<.001)	0.423 (<.001)	0.903	0.813	0.764	0		Reserved	
F14	2.366^a^ (.02)	0.141^a^ (.02)	0.909^a^	0.574	0.475^a^	4		Removed	
F15	5.106 (<.001)	0.417 (<.001)	0.903	0.776	0.796	0		Reserved	
F16	5.151 (<.001)	0.401 (<.001)	0.903	0.684	0.736	0		Reserved	
F17	–1.517^a^ (.13)	–0.142^a^ (.02)	0.914^a^	0.361	–0.505^a^	4		Removed	
F18	4.938 (<.001)	0.359^a^ (<.001)	0.904	0.755	0.795	1		Reserved	
F19	2.705^a^ (.008)	0.248^a^ (<.001)	0.906^a^	0.530	0.669	3		Removed	
F20	–0.128^a^ (.90)	–0.029^a^ (.63)	0.911^a^	0.637	–0.297^a^	4		Removed	
F21	1.536^a^ (.13)	0.124^a^ (.04)	0.909^a^	0.723	0.035^a^	4		Removed	
F22	18.411 (<.001)	0.739 (<.001)	0.896	0.669	0.711	0		Reserved	
F23	15.496 (<.001)	0.718 (<.001)	0.897	0.772	0.685	0		Reserved	
F24	11.675 (<.001)	0.641 (<.001)	0.899	0.792	0.612	0		Reserved	
F25	16.826 (<.001)	0.749 (<.001)	0.896	0.796	0.718	0		Reserved	
F26	11.327 (<.001)	0.639 (<.001)	0.899	0.821	0.615	0		Reserved	
F27	14.129 (<.001)	0.710 (<.001)	0.897	0.834	0.685	0		Reserved	
F28	13.209 (<.001)	0.677 (<.001)	0.898	0.814	0.668	0		Reserved	
Criteria	≥3.000 (N/A^b^)	≥0.400 (N/A)	≤0.904	≥0.200	≥0.600	N/A		N/A	

^a^Substandard values.

^b^N/A: not applicable.

### Face Validity of the KAP-EI Scale

The corrected I-CVI was 0.851, S-CVI/Ave was 0.901, and IRR was 0.882.

### EFA of the KAP-EI Scale

The Bartlett test was sensitive(*χ^2^*/*df* ratio=6583.040; *P*<.001), and the KMO measure of sampling adequacy for the scale was 0.930, indicating that factor analysis was applied to the scale sample. In the preliminary results, 3 factors were extracted and explained 76.76% of the variance. EFA resulted in 3 factors with a total of 23 items. The eigenvalue of factor 1, which explained 44.25% of the variance, was 10.177. Based on the scale items’ content, factor 1 was “knowledge” and comprised 10 items. The eigenvalue of factor 2, which explained 19.952% of the variance, was 4.589. Based on the scale items’ content, factor 2 was “attitude” and comprised 6 items. The eigenvalue of factor 3, which explained 12.562% of the variance, was 2.889. Based on the scale items’ content, factor 3 was “practice” and comprised 7 items (Table 3).

**Table 3:** Exploratory factor analysis of the Knowledge-Attitude-Practice in Ethics Implementation scale.

Item	Value of factor loading after rotation				Value of community
	Knowledge	Practice	Attitude		
F7	0.884	N/A^a^	N/A	0.848	
F5	0.873	N/A	N/A	0.846	
F9	0.873	N/A	N/A	0.824	
F8	0.872	N/A	N/A	0.816	
F4	0.857	N/A	N/A	0.791	
F6	0.851	N/A	N/A	0.809	
F3	0.850	N/A	N/A	0.762	
F2	0.817	N/A	N/A	0.712	
F10	0.799	N/A	N/A	0.726	
F1	0.677	N/A	N/A	0.507	
F26	N/A	0.882	N/A	0.809	
F27	N/A	0.879	N/A	0.834	
F24	N/A	0.875	N/A	0.789	
F28	N/A	0.864	N/A	0.810	
F25	N/A	0.841	N/A	0.795	
F23	N/A	0.839	N/A	0.771	
F22	N/A	0.723	N/A	0.657	
F13	N/A	N/A	0.889	0.798	
F15	N/A	N/A	0.883	0.799	
F12	N/A	N/A	0.878	0.802	
F18	N/A	N/A	0.867	0.754	
F11	N/A	N/A	0.833	0.704	
F16	N/A	N/A	0.825	0.690	
Percentage of variance, %	44.246	19.952	12.562	N/A	
Percentage of the cumulative, %	44.246	64.198	76.759	N/A	

^a^N/A: not applicable.

### CFA of the KAP-EI Scale

**Figure 1:**
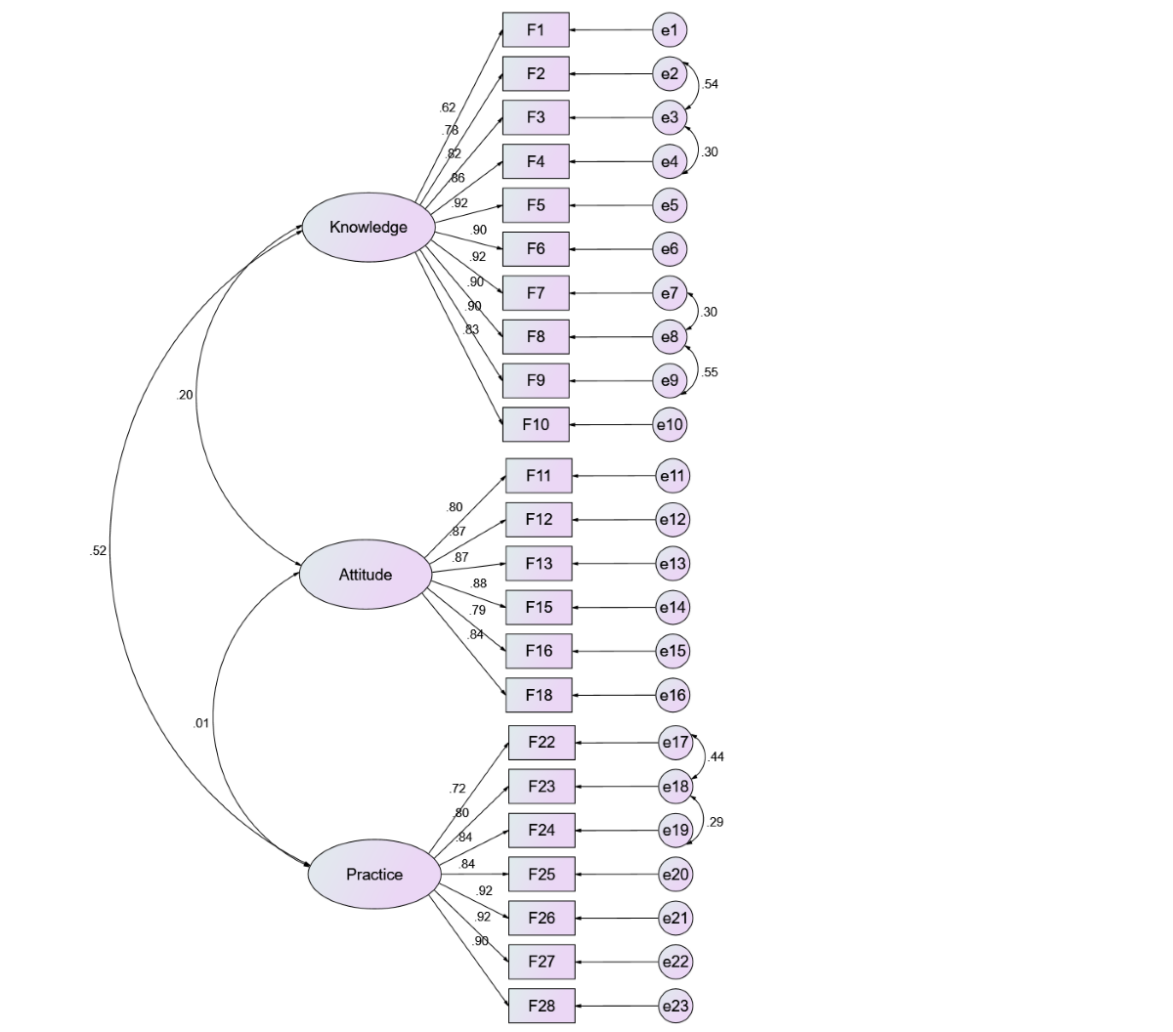
Standardized estimates of confirmatory factor analysis for the validation sample.

Fit indices with a revised parameter specification yielded better and reasonably good fit (*χ^2^*/*df* ratio=2.338, CFI=0.949, TLI=0.941, RMSEA=0.064, and SRMR=0.052), which supports the KAP-EI scale's 3D structure. Figure 1 shows the standardized estimates of CFA. The standardized factor loadings (λ) of all the items ranged from 0.62 to 0.92. The CR of the 3 dimensions of knowledge, attitude, and practice was 0.963, 0.935, and 0.948, and the average variance extracted was 0.725, 0.706, and 0.724, respectively.

### Reliability of the KAP-EI Scale

Each of the 3 dimensions demonstrated satisfactory internal consistency with Cronbach α values in the range of .935-.964. The Cronbach α for the whole scale approached .934 (Table 4).

**Table 4:** Internal consistency of the Knowledge-Attitude-Practice in Ethics Implementation (KAP-EI) scale.

Dimensions	Items, n	Score, mean (SD)	Cronbach α
Knowledge	10	29.85 (7.412)	.964
Attitude	6	23.98 (4.321)	.935
Practice	7	18.36 (7.362)	.950
KAP-EI scale	23	72.18 (1)4.132	.934

## Discussion

### Principal Findings

The purpose of this study was to develop a KAP model–based scale for researchers to measure the implementation of ethics into medical AI research and explore its validity and reliability. The Cronbach α, the value for split-half reliability and test-retest reliability of the whole scale, was higher than .7, which indicated that the scale had excellent reliability. Additionally, the corrected I-CVI was higher than 0.8, the S-CVI/Ave was more than 0.9, and IRR was more than 0.7, which implied that it had good content validity. The 3-factor model obtained after EFA was tested by CFA. Based on the results, the model (23 items) was a good fit for the data. For the item analysis, except for F12, F14, and F17-F21, the correlation coefficient between the items was greater than 0.4; the absolute value of the critical ratio of F14, F17, F19, F20, and F21 was less than 3; the revised Cronbach α coefficient after deleting F14, F17, F20, and F21 was less than the original Cronbach α coefficient (.904); and the absolute values of factor loading of F14, F17, F20, and F21 were less than 0.6. According to the experts' opinions from the focus group interview at the stage of item generation, these items were good questions because the response to either of them was uncertain and the participants might freely express their views. Notably, we intended to develop a scale to measure medical AI researchers' knowledge, attitude, and practice in the implementation of ethics, instead of investigating their views about knowledge, attitude, and practice. The results also show that questions about views were not suitable for a scale. F14, F17, and F18-F21 were deleted in this procedure. To optimize the questionnaire design, the content of items should have been more specific and directional, but we failed to do that precisely because China lacks the necessary mechanism guarantee for ethics engagement and extensive research on the very subject.

### Evaluation of the Implementation of Ethics in Medical AI

While some strategies include ways of evaluating the implementation of ethics [22,24-27], we could not find clear measures on whether the implementations were successful in medical AI research. The absence of clearly defined measures of successful implementation of ethics might reveal a lack of maturity in this emerging field. The complexity of implementing ethics may reinforce the need for a common language among different stakeholders [51].

The relationship between ethical values and behavior has attracted the interest of social scientists for several decades [52,53]. This is also reflected in correlation coefficients between the dimensions of attitude and practice. Values (referring to attitudes) are defined as desirable goals that act as guiding principles in implementing ethics. They are then translated and become visible through individual behaviors and concrete actions (referring to practice). Values might be incommensurable, and people may confer different significance to the same value [54], which indicates a weak relationship between attitude and practice in the implementation of ethics. Similarly, correlation coefficients between the dimensions of knowledge and practice also indicated a weak relationship.

Unsatisfactory performance of knowledge and practice in the implementation of ethics [55,56] may be due to the unclear ethics framework and the lack of a series of support such as ethical training [23]. As far as ethical evaluation is concerned, there are 2 important premises to decide whether the effect of ethics implementation is good or not and whether it may be reflected from medical AI researchers' KAP. One premise is the supporting mechanism, and the other is a feasible ethics framework. The supporting mechanism includes soft constraints (such as ethical norms, specific requirements for ethical review, etc), hard constraints (such as relevant laws and regulations), and a series of conditions to guarantee them. The ethics framework results from interdisciplinary cooperation of science, engineering, and ethics to some extent, which requires joint research and discussion with philosophers, medical AI researchers, end users, and policy makers. Therefore, further research to investigate the effectiveness of the framework is needed, similar to this study, to serve as evidence for decision-making. We hope that this scale serves as the first tool to embark on further medical AI ethics implementation, which still has a long way to go in China.

### Limitations

Our results raise a number of issues, which could be best explored in future research. First, the members of the expert group who participated in the stage of item generation were from the same university and the same tertiary hospital in Shanghai; thus, the geographical limitation restricts the extent of generalization of our findings. Second, the participants were recruited through snowball sampling, which might have resulted in a sample selection bias. In addition, with the rapid development of medical AI, the approaches to implementing ethics are also constantly changing. The usage of the scale contents will be limited, and it needs to be revised regularly for optimization.

### Conclusions

Creating a comprehensive scale is of paramount importance in investigating medical AI researchers' knowledge, attitudes, and practices of ethics implementation. The KAP-EI scale appears to be a reliable and valid instrument developed to advance the measurement of the perception of implementing ethics among medical AI researchers. To our knowledge, the KAP-EI scale is the first instrument designed for this purpose.

## Appendix

Multimedia Appendix 1 Data sources, retrieval strategies, and primary reference lists.

Multimedia Appendix 2 Final draft of the KAP-EI scale. KAP-EI: Knowledge-Attitude-Practice in Ethics Implementation.

Multimedia Appendix 3 Final version of the KAP-EI scale. KAP-EI: Knowledge-Attitude-Practice in Ethics Implementation.

## Abbreviations

AI: artificial intelligence
CFA: confirmatory factor analysis
CFI: comparative fit index
COSMIN: e COnsensus-based Standards for the selection of health status Measurement INstruments
CR: critical ratio
EFA: exploratory factor analysis
I-CVI: item-level content validity index
IRR: interrater reliability
KAP: Knowledge-Attitude-Practice
KAP-EI: Knowledge-Attitude-Practice in Ethics Implementation
KMO: Kaiser-Meyer-Olkin
RMSEA: root-mean-square error of approximation
S-CVI/Ave: average scale–level content validity index
SRMR: standardized root-mean-square residual
TLI: Tucker Lewis index

## Data Availability

The data sets generated during or analyzed during this study are not publicly available due to hospital policy but are available from the corresponding author on reasonable request.
