# Supplementary material for: Development, Reliability, and Structural Validity of the Scale for Knowledge, Attitude, and Practice in Ethics Implementation Among AI Researchers: Cross-Sectional Study
Source: JMIR Form Res. 2023 Oct 26;7:e42202. doi: 10.2196/42202 (PMC10636617; doi:10.2196/42202)
Supplement: Multimedia Appendix 1 [file formative_v7i1e42202_app1.docx]

**Multimedia Appendix 1:**

1 Data Source

Databases: BYU Law, Westlaw, Web of Science, JSTOR, Springer

International Organization Website: UNESCO Library, OECD Library, EP Library, et al.

national government departments and relevant committees :Using the United States as an example: THE WHITE HOUSE(http://www.whitehouse.gov), Science technology council(https://www.nstc.org.zm), homeland security(http://www.dhs.gov), Information Network Sector(http://www.nitrd.gov), department of Defense (https://innovation.defense.gov/).

2 Retrieval Strategy

The title should contain: (artificial intelligence or AI or robot) and (ethic or moral or governance or risk or principle or guideline or consensus)

Time Range: January 1, 2016 to May 1, 2021

3 Primary Reference Lists

| NO. | Publishing Agency | Document Title | Year |
| --- | --- | --- | --- |
| 1 | European Parliament | An EU framework for artificial intelligence | 2020 |
| 2 | European Parliament | Artificial intelligence: From ethics to policy | 2020 |
| 3 | European Parliament | European framework on ethical aspects of artificial intelligence, robotics and related technologies | 2020 |
| 4 | European Parliament | EU guidelines on ethics in artificial intelligence: Context and implementation | 2019 |
| 5 | European Parliament | European Civil Law Rules in Robotics | 2016 |
| 6 | European Commission | Ethics Guidelines for Trustworthy AI | 2018 |
| 7 | OECD | OECD Principles on AI | 2019 |
| 8 | China | 网络安全标准实践指南—人工智能伦理安全风险防范指引》 | 2021 |
| 9 | China | 《新一代人工智能治理原则——发展负责任的人工智能》 | 2019 |
| 10 | UNESCO | Recommendation on the Ethics of Artificial Intelligence | 2021 |
| 11 | UNESCO | Preliminary study on the Ethics of Artificial Intelligence | 2019 |
| 12 | UNESCO | Ethical principles for the development of Artificial Intelligence based on the diversity of cultural expressions | 2018 |
| 13 | America | Executive Order Promoting the Use of Trustworthy AI in the Federal Government | 2020 |
| 14 | America | Technology Assessment: Artificial Intelligence in Health Care | 2020 |
| 15 | America | Artificial Intelligence Ethics Framework for the Intelligence Community | 2020 |
| 16 | America | Four Principles of Explainable Artificial Intelligence | 2020 |
| 17 | America | Ethical Principles for Artificial Intelligence | 2020 |
| 18 | America | Principles of Artificial Intelligence Ethics for the Intelligence Community | 2020 |
| 19 | America | Guidance for Regulation of Artificial Intelligence Applications | 2020 |
| 20 | Britain | Robots and robotic devices Guide to the ethical design and application of robots and robotic systems | 2016 |
| 21 | The European Union | Ethics Guidelines for Trustworthy AI | 2020 |
| 22 | Korea | National Ethical Standards for Artificial Intelligence | 2020 |
| 23 | WHO | Ethics and governance of artificial intelligence for health: WHO guidance | 2021 |
| 24 | Australia | Australia’s Artificial Intelligence Ethics Framework | 2019 |
| 25 | IEEE | Ethical Guidelines for the Design of Artificial Intelligence | 2019 |
| 26 | IEEE | Ethically Aligned Design Version 2 | 2017 |
| 27 | Singapore | Model AI Governance Framework | 2019 |
