# Supplementary material for: Development, Reliability, and Structural Validity of the Scale for Knowledge, Attitude, and Practice in Ethics Implementation Among AI Researchers: Cross-Sectional Study
Source: JMIR Form Res. 2023 Oct 26;7:e42202. doi: 10.2196/42202 (PMC10636617; doi:10.2196/42202)
Supplement: Multimedia Appendix 3 [file formative_v7i1e42202_app3.docx]

**医疗人工智能研究伦理的知信行量表**

**（一）认知维度**

**您对下面条目所描述内容的了解程度怎样？请选出最符合自己实际情况的选项。**

1 人工智能技术在医疗领域的应用现状。

□完全不了解

□不太了解

□一般

□比较了解

□完全了解

2 医疗人工智能研发/应用中的伦理问题。

□完全不了解

□不太了解

□一般

□比较了解

□完全了解

3 医疗人工智能研发/应用相关伦理问题的成因。

□完全不了解

□不太了解

□一般

□比较了解

□完全了解

4 医疗人工智能研发/应用中伦理问题的应对策略。

□完全不了解

□不太了解

□一般

□比较了解

□完全了解

5 医疗人工智能研发/应用的伦理原则/规范。

□完全不了解

□不太了解

□一般

□比较了解

□完全了解

6 医疗人工智能研发/应用的伦理政策/法规。

□完全不了解

□不太了解

□一般

□比较了解

□完全了解

7 医疗人工智能研发/应用的伦理审查内容。

□完全不了解

□不太了解

□一般

□比较了解

□完全了解

8 医疗人工智能研发/应用的伦理监管机制。

□完全不了解

□不太了解

□一般

□比较了解

□完全了解

9 医疗人工智能研发/应用的伦理风险管理。

□完全不了解

□不太了解

□一般

□比较了解

□完全了解

10 医疗人工智能研发/应用中出现伦理失范引发的后果。

□完全不了解

□不太了解

□一般

□比较了解

□完全了解

**（二）态度维度**

**您对下面条目所描述内容的认同程度怎样？请选出最符合自己实际想法的选项：**

11科研伦理（审查）委员会加入人工智能相关知识背景的专家方可对医疗人工智能项目做出全面的评估和审查。

□完全不赞同

□不太赞同

□一般

□比较赞同

□完全赞同

12医疗人工智能的伦理原则/规范需转译为易于理解、可操作的工作流程才能落地执行。

□完全不赞同

□不太赞同

□一般

□比较赞同

□完全赞同

13邀请伦理专家参与医疗人工智能研发/应用有助于项目的伦理管理。

□完全不赞同

□不太赞同

□一般

□比较赞同

□完全赞同

14 明确医疗人工智能研发/应用的伦理责任主体有益于伦理管理。

□完全不赞同

□不太赞同

□一般

□比较赞同

□完全赞同

15 参加专门的医疗人工智能伦理教育与培训对实践项目的伦理管理有帮助。

□完全不赞同

□不太赞同

□一般

□比较赞同

□完全赞同

16 建立切实有效的监管机制有助于医疗人工智能研发/应用的伦理管理。

□完全不赞同

□不太赞同

□一般

□比较赞同

□完全赞同

**（三）行为维度**

**在您主持/参与医疗人工智能研发/应用项目时，是否实施过以下行为？如果是，那么每项行为在您所主持/参与的所有医疗人工智能研究项目中出现的频率怎样？请选出最符合自己实际情况的选项：**

17 将项目方案提交科研伦理（审查）委员会审查批准[去了解项目的伦理审查（批准）信息]。

□从未

□很少

□有时

□经常

□总是

18跟踪研判项（或去了解）目的伦理风险。

□从未

□很少

□有时

□经常

□总是

19发布或接受项目的伦理风险预警信息。

□从未

□很少

□有时

□经常

□总是

20 与所有项目参与人员沟通项目的伦理管理方案(去了解项目的伦理管理措施)。

□从未

□很少

□有时

□经常

□总是

21 组织演练或参与培训项目的伦理失范处置预案。

□从未

□很少

□有时

□经常

□总是

22 评价或反馈项目伦理管理方案的实施效果。

□从未

□很少

□有时

□经常

□总是

23 接受专门的医疗人工智能伦理教育与培训（或组织项目人员参与专门的医疗人工智能伦理教育与培训）。

□从未

□很少

□有时

□经常

□总是
